# Interprofessional Education in Saudi Arabia: A Call to Action

**DOI:** 10.1055/s-0044-1788321

**Published:** 2024-07-16

**Authors:** Ahmed Yaseen Alqutaibi, Rawda Awad, Mohamed M. Rahhal, Muhammad S. Zafar

**Affiliations:** 1 Substitutive Dental Sciences Department (Prosthodontics), College of Dentistry, Taibah University, Al Madinah, Saudi Arabia; 2 Department of Prosthodontics, College of Dentistry, Ibb University, Ibb, Yemen; 3 Department of Restorative Dentistry, Missouri School of Dentistry and Oral Health (MOSDOH), A.T. Still University, Missouri, United States; 4 Department of Prosthodontics, Faculty of Oral and Dental Medicine, Fayoum University, Fayoum, Egypt; 5 Department of Restorative Dentistry, Taibah University, Al Madinah Al Munawwarah, Saudi Arabia; 6 Center of Medical and Bio-allied Health Sciences Research, Ajman University, Ajman, United Arab Emirates; 7 Department of Dental Materials, Islamic International Dental College, Riphah International University, Islamabad, Pakistan

Health care education in Saudi Arabia, like many other regions worldwide, faces various challenges due to the complex medical landscape. The need for a health care workforce is proficient in interprofessional collaboration is crucial in addressing these challenges. Despite the widely recognized importance of interprofessional education (IPE) globally, its integration into the curricula of health science schools in Saudi Arabia is still in its early stages. This editorial aims to highlight the deficiencies of IPE in Saudi Arabia, identify existing barriers, and propose strategic measures to overcome these challenges and successfully incorporate IPE.

The health care sector in Saudi Arabia is currently experiencing a swift transformation characterized by an increasing focus on enhancing health care outcomes through collaborative practice. The Vision 2030 initiative highlights the significance of improving health care services and establishing a sustainable health care system. IPE is pivotal in attaining these objectives by cultivating a collaborative workforce capable of providing top-notch, patient-centered care. Although the concept of IPE is not groundbreaking in Western contexts, its integration into the curricula of medical and health sciences education in Saudi Arabia has been relatively recent and limited. The establishment of IPE initiatives has been irregularly documented, with a small number of studies 1 2 indicating a positive response to these initiatives. However, the overall implementation across the kingdom remains slow and fragmented. The existing IPE framework in Saudi Arabia lacks a standardized approach, and there is a requirement for a well-defined curriculum that incorporates tailored teaching and learning methods and assessment strategies that are relevant to the local context.

The shortcomings evident in implementing IPE in Saudi Arabia can be ascribed to various factors such as cultural resistance, institutional inertia, faculty development, logistical challenges, and limited research. 3 Cultural resistance emerges as a noteworthy contributing factor, as the shift from a traditional segregated learning model to a collaborative educational approach frequently faces opposition deeply ingrained in cultural norms that prioritize hierarchical structures and professional autonomy. Furthermore, institutional inertia presents a significant obstacle; academic and health care establishments may grapple with the perceived intricacy of revamping current curricula and uncertainties surrounding the outcomes associated with integrating IPE. 3 Furthermore, it is important to note that there is a significant scarcity of faculty members who possess the necessary expertise to instruct and evaluate IPE skills effectively. This shortage obstructs the implementation of IPE programs. In addition, logistical obstacles present considerable challenges. These include the coordination of timetables among diverse professional programs, the identification of appropriate learning settings, and the creation of interdisciplinary case studies. Finally, the dearth of localized research on IPE in Saudi Arabia acts as a pivotal barrier to comprehending its impact and customizing programs to cater to the unique requirements of the region. 3

To address these limitations and establish the foundation for the successful integration of IPE into health science curricula, it is imperative to consider several strategic initiatives. The involvement of stakeholders is of utmost importance; engaging a diverse range of stakeholders, such as governmental health departments, educational institutions, accreditation bodies, and health care providers, can contribute to the establishment of a shared vision for IPE. 4 Cultural sensitivity is also crucial; the implementation of change management strategies that incorporate cultural sensitization programs can adequately prepare all stakeholders for the transition to a more collaborative educational paradigm. 5 Moreover, the development of an interprofessional curriculum that emphasizes shared learning objectives and outcomes applicable to all health science students is indispensable to fostering a cohesive learning environment. 6 Investing in faculty training programs is a crucial step in equipping educators with the necessary skills and knowledge to facilitate and assess IPE effectively. 4 Moreover, implementing pilot IPE programs allows for continuous learning and adjustments before full integration into the curricula while conducting localized research and establishing regular feedback mechanisms ensures ongoing improvement. 6 Adequate allocation of resources and the establishment of infrastructure that supports interprofessional learning, such as collaborative learning spaces and shared simulation centers, are essential. 4 Finally, collaborating with accreditation bodies to develop policies that endorse and require the inclusion of IPE in health sciences education will provide a strong policy framework to support these endeavors.

In conclusion, the successful integration of IPE within the health science schools of Saudi Arabia signifies more than a mere educational reform; instead, it reflects a significant paradigm shift toward a more collaborative and efficient health care system. Although substantial challenges can be overcome, they can be strategically and culturally sensitively addressed. The benefits of this transformative process, such as a well-equipped workforce capable of providing excellent, patient-centered care, justify the investment and exertion required. All stakeholders need to acknowledge the urgency of this educational transformation and unite their efforts to establish a future health care system characterized by collaboration.
